# From kitchen to classroom: Assessing the impact of cleaner burning biomass-fuelled cookstoves on primary school attendance in Karonga district, northern Malawi

**DOI:** 10.1371/journal.pone.0193376

**Published:** 2018-04-12

**Authors:** Christine A. Kelly, Amelia C. Crampin, Kevin Mortimer, Albert Dube, Jullita Malava, Deborah Johnston, Elaine Unterhalter, Judith R. Glynn

**Affiliations:** 1 Faculty of Epidemiology and Population Health, London School of Hygiene and Tropical Medicine, London, United Kingdom; 2 Malawi Epidemiology and Intervention Research Unit, Chilumba/Lilongwe, Malawi; 3 Department of Clinical Sciences, Liverpool School of Tropical Medicine, Liverpool, United Kingdom; 4 Malawi Liverpool Wellcome Trust Programme, Blantyre, Malawi; 5 Department of Economics, SOAS, University of London, London, United Kingdom; 6 Department of Education, Practice and Society, UCL Institute of Education, London, United Kingdom; Massachusetts General Hospital, UNITED STATES

## Abstract

Household air pollution from burning solid fuels is responsible for an estimated 2.9 million premature deaths worldwide each year and 4.5% of global disability-adjusted life years, while cooking and fuel collection pose a considerable time burden, particularly for women and children. Cleaner burning biomass-fuelled cookstoves have the potential to lower exposure to household air pollution as well as reduce fuelwood demand by increasing the combustion efficiency of cooking fires, which may in turn yield ancillary benefits in other domains. The present paper capitalises on opportunities offered by the Cooking and Pneumonia Study (CAPS), the largest randomised trial of biomass-fuelled cookstoves on health outcomes conducted to date, the design of which allows for the evaluation of additional outcomes at scale. This mixed methods study assesses the impact of cookstoves on primary school absenteeism in Karonga district, northern Malawi, in particular by conferring health and time and resource gains on young people aged 5–18. The analysis combines quantitative data from 6168 primary school students with in-depth interviews and focus group discussions carried out among 48 students in the same catchment area in 2016. Negative binomial regression models find no evidence that the cookstoves affected primary school absenteeism overall [IRR 0.92 (0.71–1.18), p = 0.51]. Qualitative analysis suggests that the cookstoves did not sufficiently improve household health to influence school attendance, while the time and resource burdens associated with cooking activities—although reduced in intervention households—were considered to be compatible with school attendance in both trial arms. More research is needed to assess whether the cookstoves influenced educational outcomes not captured by the attendance measure available, such as timely arrival to school or hours spent on homework.

## Introduction

Ninety-five percent of households in Malawi rely on biomass fuels such as wood, charcoal or crop residues for cooking [1], often in poorly ventilated environments. Household air pollution from solid cookfuels (HAP) is responsible for an estimated 2.9 million premature deaths worldwide each year from causes including pneumonia, stroke, ischaemic heart disease, chronic obstructive pulmonary disease, and lung cancer [2, 3], and contributes 4.5% of global disability-adjusted life years [4]. Among school-age children specifically, there is some evidence of an association between HAP and acute respiratory infections as well as asthma [5–7].

Cleaner burning biomass-fuelled cookstoves, which have better fuel efficiency than traditional open fire cooking methods and reduce harmful emissions, have been advocated as a means to reduce morbidity and mortality associated with cooking with solid fuels. Outcomes of existing randomised controlled trials, however, have principally targeted women and young children, as the groups with the highest exposure to solid fuel emissions, and have typically not explored the cookstoves’ impact on other household members, particularly adolescents. Moreover, less is known about the potential ancillary benefits of cookstoves in domains beyond health [8, 9]. The present study fills an important research gap by assessing the effect of cleaner burning biomass-fuelled cookstoves on school attendance of young people in northern Malawi.

**Fig 1:**
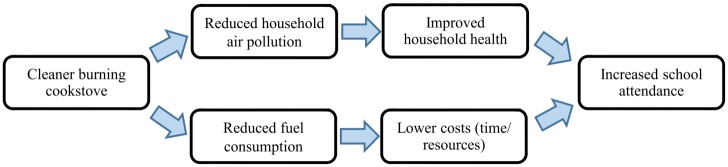
Proposed causal pathways linking cleaner burning cookstoves with increased school attendance.

Two possible mechanisms are explored: 1) reduced household air pollution leading to improved health and associated reductions in caregiving responsibilities, and 2) reduced fuel consumption leading to lower time and resource costs associated with acquiring fuel, both yielding increased school attendance (Fig 1). Although the impact of cooking-related activities on school attendance has not been formally assessed in sub-Saharan Africa, the contributions of ill health and caregiving to absenteeism are well established. Research from southern Nigeria has specifically highlighted respiratory illness as a reason for absenteeism [10]. The authors found that 2.5% of children aged 7–14 years, including 5.7% of children in rural areas, reported missing school in the past twelve months due to symptoms of respiratory illness, although episodes were not necessarily linked to HAP exposure.

Cleaner burning cookstoves may also reduce school absenteeism by improving the health of other household members. Several studies from sub-Saharan Africa have highlighted the responsibility schoolchildren bear for providing care or performing household or economic labour in the event of a family illness, to the detriment of their school attendance. Research from Ethiopia showed that, controlling for sociodemographic factors, high levels of absenteeism were significantly associated with the percentage of household members who were sick for more than 30 days in the previous year [11]. Analyses of orphanhood in Kenya and Tanzania found that children’s school attendance declined not only in the wake of a parental death, but also in the months leading up to it, presumably as students served as caregivers [12, 13]. Interviews from a mixed methods study in South Africa indicated that adolescents missed school to accompany ill relatives to health facilities or provide home-based care [14]. Thus, potential health improvements for both school-age children and other household members through reduced exposure to cooking-related pollutants could yield significant payoffs with respect to school attendance.

The second pathway focuses on time and resource factors linking cleaner burning cookstoves with reduced absenteeism. Even in healthy households, students regularly participate in domestic or market activities that can compete with school attendance. Data from UNICEF Multiple Indicator Cluster Surveys in Malawi show that boys and girls spend an average of 9 and 12 hours per week, respectively, on household work, and an additional 3 and 4 hours on family business work [15]. Water and fuelwood collection have been identified as particularly burdensome for school-going children [16]. A mixed methods study across 24 sites in Ghana, Malawi and South Africa suggested that the time and exertion associated with child porterage—carrying water, firewood, and agricultural produce—as well as the prospect of earning extra money from commercial load carrying, contributed to tardiness and absenteeism [17]. By reducing the duration and/or frequency of domestic fuelwood collection, as well as speeding up cooking times of household meals, cleaner burning cookstoves may thus play an important role in improving school attendance. Moreover, although the vast majority (94.8%) of households in Karonga district, Malawi, collect their own firewood for cooking, 4.6% purchase their wood supplies [18]. Reduced expenditure on firewood associated with cleaner burning cookstoves may enable households to better meet schooling costs, including exercise books, pens and clothes, which have been shown to amount to approximately 6% per child of the total financial resources of the poorest Malawian households [19].

This paper capitalises on opportunities offered by the Cooking and Pneumonia Study (CAPS), the largest randomised trial of cleaner burning biomass-fuelled cookstoves on health outcomes conducted to date, to assess the impact of cookstoves on primary school absenteeism in Karonga district, northern Malawi. By combining quantitative data from 6168 primary school students collected under the auspices of the Karonga Health and Demographic Surveillance System (HDSS) with in-depth interviews and focus group discussions carried out among 48 students in the same catchment area, it compares school absenteeism levels across trial arms as well as explores the underlying mechanisms linking cookstoves with school attendance.

## Methods

### Ethics statement

Data collection associated with the Cooking and Pneumonia Study was reviewed and approved by the Malawi College of Medicine Research and Ethics Committee (P.11/12/1308) and the Liverpool School of Tropical Medicine Research Ethics Committee (Ref #12.40). Data collection associated with the Karonga Health and Demographic Surveillance System was reviewed and approved by the National Health Sciences Research Committee (NHSRC) in Malawi (Protocol #419) and the London School of Hygiene and Tropical Medicine (LSHTM) Research Ethics Committee (Ref #5081). Data collection associated with the nested qualitative study was reviewed and approved by the NHSRC in Malawi (Protocol #15/11/1509) and the LSHTM Research Ethics Committee (Ref #10401).

### Trial design

The Cooking and Pneumonia Study, implemented from July 2014-September 2016, involved one hundred community-level clusters in Karonga district, northern Malawi, randomised to intervention or control groups. An additional 50 clusters were enrolled in a second site in southern Malawi, but these are not the focus of the present study. A full description of the trial design and randomisation procedures is available in Mortimer et al. [20]. Within intervention clusters, eligible households received two Philips HD4012LS cookstoves with cooking pots and a solar panel with which to charge the in-built battery-powered fan, as well as user training. As the trial’s primary outcome of interest was incidence of pneumonia in children under five years old [20], cookstoves were distributed only to households with children below 4.5 years at baseline, as well as on a continuous basis to eligible in-migrating households or those into which children under five were born, adopted or fostered over the course of the two-year follow-up period. The CAPS team visited households approximately every three months to collect information about cookstove usage and functionality. A free repair, maintenance and replacement service was provided for damaged cookstoves and solar panels. Control households received their own cookstoves at the end of the trial.

### Study population

To examine the impact of cookstoves on primary school attendance, we identified young people of primary school age resident in households enrolled in the CAPS trial. Primary school in Malawi comprises eight grades but, in light of the frequency of late entry and grade repetition among Malawian students [21], we included children aged 5–18. The total number of absence days in the past four weeks that school was in session was collected as part of the annual household survey of the Karonga Health and Demographic Surveillance System [22], which formed the catchment area of the CAPS trial. School attendance reports were drawn from the first HDSS interview that took place at least 60 days after the household was enrolled in CAPS. A threshold of 60 days was chosen to allow for a short cookstove adjustment period—for instance, to deplete existing stocks of firewood—and in light of the HDSS survey design in which absenteeism over the past four weeks was retrospectively reported. Schooling information was provided by household members aged 15 years or older who were at home at the time of the field team’s visit; as such, most respondents were parents or other adult relatives reporting on behalf of resident children.

The primary analysis followed intention-to-treat (ITT) principles, where the ITT population consisted of primary school students aged 5–18 living in CAPS intervention or control households at the time of enrolment, and who had at least one follow-up CAPS and HDSS survey. A per-protocol analysis was also conducted for comparison, excluding students who changed cookstove exposure status between CAPS enrolment and the first eligible HDSS survey by: 1) moving from an intervention household to a new household in a control cluster, 2) moving from a control household to a new intervention household, or 3) moving from a cookstove to a non-cookstove household within an intervention cluster. It also excluded students living in households that reported not using the cookstove exclusively in the CAPS visit closest to the HDSS schooling interview—that is, households that did not use the cookstove as a result of breakage, mechanical failure, or personal preference, or that continued to use open fire cooking methods alongside the cookstove for at least some household meals. Finally, the per-protocol analysis excluded students from households for whom data from a CAPS follow-up visit were not available within three months of the HDSS survey.

### Statistical methods

Negative binomial regression modelling was used to compare absenteeism across trial groups to reflect overdispersion in the distribution of absence days. Given the high proportion of non-absence in the study population, zero-inflated negative binomial regression models were also estimated but were found neither to perform better than, nor to change the conclusions of, standard negative binomial models. All regression models included cluster robust standard errors to account for the clustered trial design. Multivariable models adjusted for the following pre-specified covariates, informed by the analyses presented in Mortimer et al. [20]: age, sex, current grade attended, repetition of current grade, maternal death, paternal death, maternal education, paternal education, total number of household members, number of younger household members, relationship to household head, sex of household head, household socioeconomic status, co-residence with a regular smoker, and exposure to sources of household smoke other than cooking. Socioeconomic status was constructed by using principal components analysis [23, 24] to generate a wealth index combining ownership of ten durable goods, two variables indicating a shortage of food or bathing soap in the past year, and two variables indicating a household’s access to an improved water source or improved toilet facility. A variable indicating the HDSS survey round was also included in regression models to control for survey-specific differences in absenteeism reporting, as was the month of interview to account for seasonal differences in absenteeism. A further variable indicating whether HDSS survey took place during term time or school holiday was additionally included, alongside a variable specifying the number of months between CAPS enrolment and the HDSS survey to adjust for potential changes in cookstove usage over time.

By adding appropriate interaction terms to each regression model, subgroup analyses were also conducted to investigate the following secondary hypotheses:

1. Cookstoves will lead to greater reductions in absenteeism for girls relative to boys, due to diminished cooking, fuel collection and caregiving responsibilities, which are predominantly carried out by girls.
2. Cookstoves will lead to greater reductions in absenteeism as children’s age increases, as older children assume more responsibility for caregiving and household chores. This analysis is guided by the age thresholds stipulated by ILO Convention No. 138 for child work burdens, namely <12, 12–14, and 15+ [25].
3. Greater reductions in absenteeism will be observed among children interviewed in the rainy season (December-April) relative to in the dry season, due to the increased propensity to cook indoors during the rainy season and the larger anticipated health benefit of using cookstoves rather than open fires in a poorly ventilated environment.

### Nested qualitative study

To corroborate the quantitative comparisons and elucidate the proposed mechanisms at play [26], a nested qualitative study was conducted in April-May 2016 among male and female primary school students aged 12–18, involving 16 in-depth interviews (IDIs) and four focus group discussions (FGDs) with eight participants per group. The qualitative sample was purposively selected using the HDSS and CAPS datasets to ensure distribution across trial arms, variation by age and school grade attended, as well as representation from the three community types present in the study area: lakeshore, roadside, and rural agricultural. Interviews and FGDs solicited students’ perceptions of the barriers to regular school attendance, household and community support for schooling, intra-household allocation of domestic responsibilities and household health status. As the basis for follow-up discussion about students’ time use, IDIs additionally included an exercise whereby participants were asked to fill a timeline with the activities in which they had engaged on the previous school day, choosing from a selection of ten illustrated activity cards: attending school, doing homework, going to the market, collecting firewood, drawing water, cooking, fishing, farming, caregiving, and playing. Among cookstove recipients, IDIs also explored the perceived impact of cleaner burning cookstoves on health, schooling and time allocation. In-depth interview and FGD topic guides were iteratively updated to reflect emerging themes from a pilot phase and from preliminary data analysis.

Qualitative activities were conducted by a team of four trained interviewers/facilitators in the participants’ local language, Chitumbuka, and subsequently transcribed and translated into English by the same research team. As a validity check, four IDI transcripts—one per interviewer—were externally audited for completeness and accuracy by a bilingual consultant. Since errors identified during this process were minimal and minor, no additional review of the remaining transcripts was undertaken. The final transcripts were uploaded into *NVivo* software for coding and thematic analysis [27], with particular focus on aspects of students’ narratives that supported, challenged or undermined the pathways linking cookstoves and school attendance shown in Fig 1.

## Results

### Analytic sample

**Fig 2:**
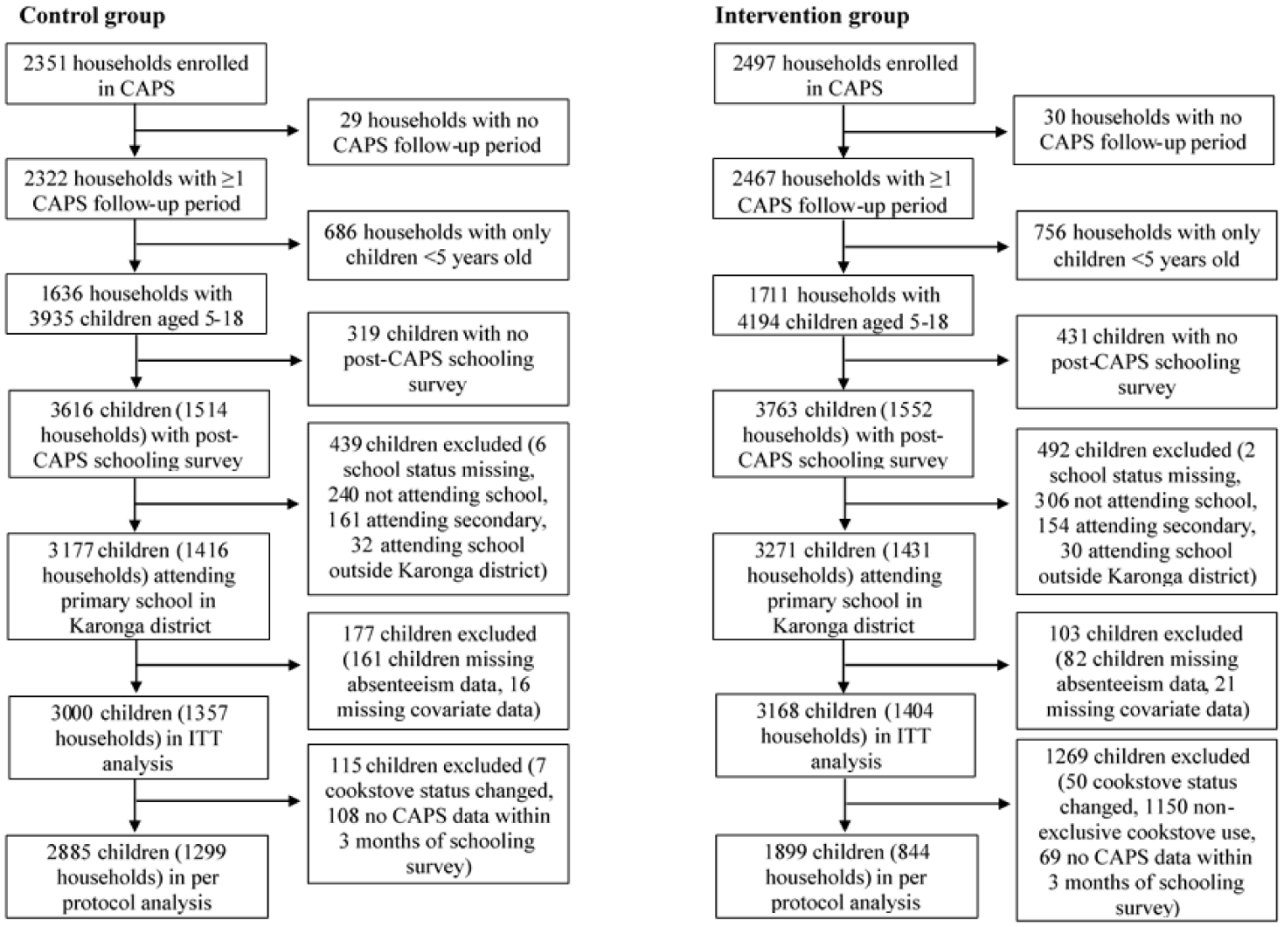
Flowchart of control and intervention participants included in analysis.

Of the 4848 households enrolled in the CAPS trial in Karonga district, 59 (30 intervention, 29 control) withdrew from the study, became ineligible, left the study area or were lost to follow-up before completing a CAPS follow-up visit. An additional 1442 (756 intervention, 686 control) had no resident school-age children. In the remaining 3347 households, 8129 young people aged 5–18 were identified (4194 intervention, 3935 control). Of these, 750 (431 intervention, 319 control) were excluded as they had no post-CAPS schooling survey before or within 30 days after the conclusion of the trial, and a further 930 (492 intervention, 438 control) were not currently attending primary school in Karonga district. Among eligible students, 281 (103 intervention, 178 control) were missing outcome or covariate data, leaving an ITT sample of 3168 and 3000 in the intervention and control groups, respectively (Fig 2). The per-protocol sample further excluded 57 children (50 intervention, 7 control) who changed cookstove status before the first schooling interview; 1150 from intervention households that reported not using the cookstove exclusively during the closest CAPS follow-up period to the schooling survey, including 92 in households that did not use the cookstove at all; and 177 (69 intervention, 108 control) for whom CAPS data within three months of the HDSS schooling survey were not available. Thus, the per-protocol sample consisted of 1899 children in intervention households and 2885 in control households (Fig 2).

### Baseline characteristics

Table 1 shows individual- and household-level baseline characteristics of the ITT sample. Household data were drawn from the CAPS baseline survey, but this did not include individual-level information about household members aged 5 or over. Hence, individual data were taken from the nearest available HDSS survey before CAPS enrolment, or up to 30 days afterward. Data were not available for 97 children who were not interviewed prior to CAPS enrolment. Additionally, since the HDSS survey and CAPS enrolment occurred on average 168 days apart (180 days intervention, 156 control), time-varying characteristics such as school enrolment and parental survival may not reflect children’s status at the start of the CAPS trial. Nevertheless, the table demonstrates that both individual- and household-level characteristics were reasonably balanced between intervention and control groups.

**Table 1:** Household- and individual-level baseline characteristics of intention-to-treat population, by trial group.

**Household characteristics**	**Control** **(N = 1357)**		**Intervention** **(N = 1404)**	
	**n**	**%**	**n**	**%**
**Sources of cooking fuel**^a^				
Firewood	1329	97.9	1383	98.5
Crop residues	804	59.2	802	57.1
Charcoal	378	27.9	307	21.9
Other	20	1.5	11	0.8
**Cooking location, dry season**				
Separate structure, roof only	314	23.1	345	24.6
Separate structure, walls and roof	706	52.0	725	51.6
Outside, open air	281	20.7	271	19.3
Outside, veranda	26	1.9	20	1.4
Inside, kitchen	20	1.5	31	2.2
Inside, living space	10	0.7	12	0.9
**Cooking location, rainy season**				
Separate structure, roof only	294	21.7	331	23.6
Separate structure, walls and roof	782	57.6	781	55.6
Outside, open air	11	0.8	16	1.1
Outside, veranda	117	8.6	113	8.0
Inside, kitchen	93	6.9	101	7.2
Inside, living space	60	4.4	62	4.4
**Sources of household smoke exposure**^a^				
Resident smoker	186	13.7	229	16.3
Burning rubbish	941	69.3	967	68.9
Cooking business	295	21.7	343	24.4
Burning bricks	137	10.1	103	7.3
Kerosene lamp	64	4.7	61	4.3
**Socioeconomic quintile**				
Lowest	277	20.4	322	22.9
2	259	19.1	299	21.3
3	254	18.7	275	19.6
4	266	19.6	258	18.4
Highest	301	22.2	250	17.8
**Individual characteristics**	**Control** **(N = 3000)**		**Intervention** **(N = 3168)**	
	**n**	**%**	**n**	**%**
**Age**				
5–11	2000	66.7	2155	68.0
12–14	641	21.4	669	21.1
≥15	359	12.0	344	10.9
Mean (years)		9.94		9.93
**Sex**				
Male	1515	50.5	1642	51.8
Female	1485	49.5	1526	48.2
**Among students with baseline interview:**	**(N = 2947)**		**(N = 3124)**	
**School status**				
Not attending	360	12.2	375	12.0
Attending standard 1–4	1856	63.0	2003	64.1
Attending standard 5–7	665	22.6	694	22.2
Attending standard 8	66	2.2	52	1.7
**Repeated current standard (if attending school)**				
Yes	672	26.0	789	28.7
No	1915	74.0	1960	71.3
**Days of absence in past 4 weeks (if attending school)**				
0	1952	75.5	2021	73.5
1	258	10.0	303	11.0
2–4	258	10.0	313	11.4
≥5	90	3.5	99	3.6
Missing	29	1.1	13	0.5
Mean days, all students		0.67		0.70
Mean days, conditional on absence		2.81		2.67
**Mother died**				
Yes	77	2.6	57	1.8
No	2870	97.4	3067	98.2
**Father died**				
Yes	193	6.5	187	6.0
No	2754	93.5	2937	94.0
**Mother’s education**				
None/primary	2362	80.1	2524	80.8
More than primary	585	19.9	600	19.2
**Father’s education**				
None/primary	1750	59.4	1945	62.3
More than primary	1197	40.6	1179	37.7
**Relationship to household head**				
Child	2422	82.2	2624	84.0
Step-child	116	3.9	116	3.7
Grandchild	261	8.9	250	8.0
Niece/nephew	53	1.8	60	1.9
Other	95	3.2	74	2.4
**Mean days between baseline and CAPS enrolment**^b^		156.4		180.6

^a^ Multiple responses possible.

^b^ Each participant’s baseline interview was assigned as the closest before CAPS enrolment or up to 30 days afterward.

### Absenteeism in the past four weeks

**Fig 3:**
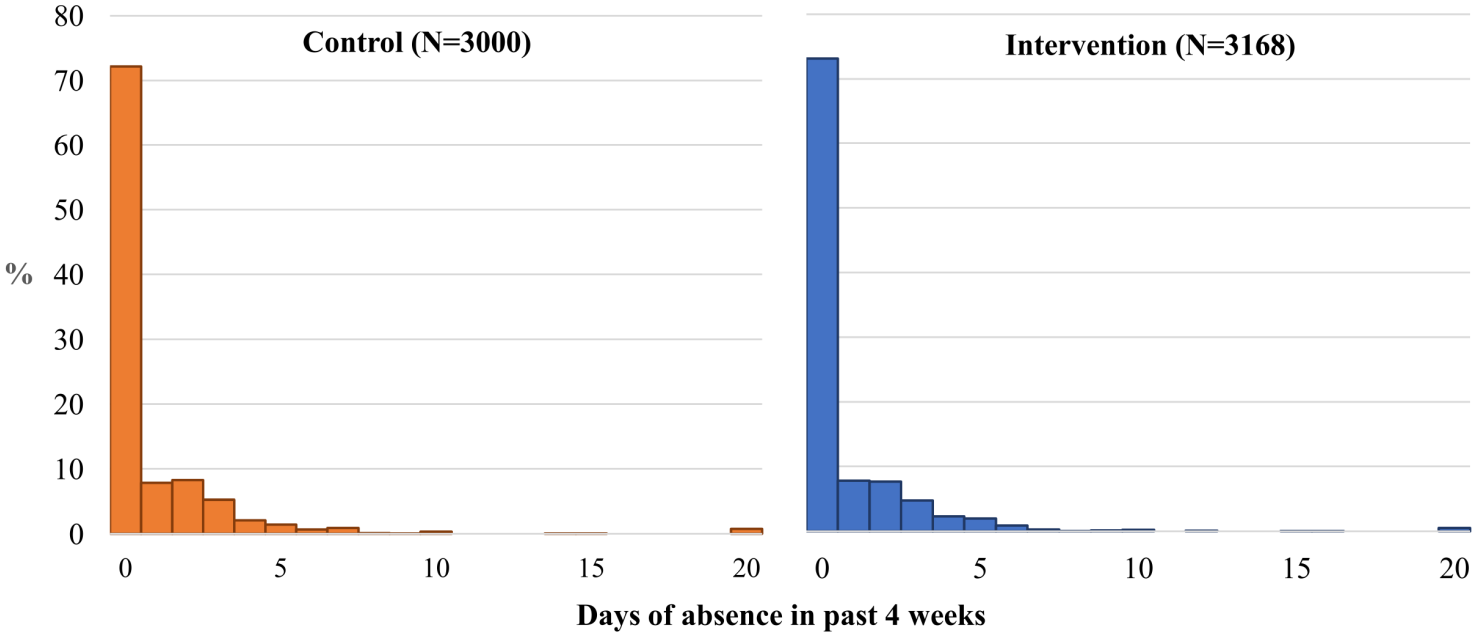
Distribution of primary school absenteeism in the past four weeks, by trial group.

**Fig 4:**
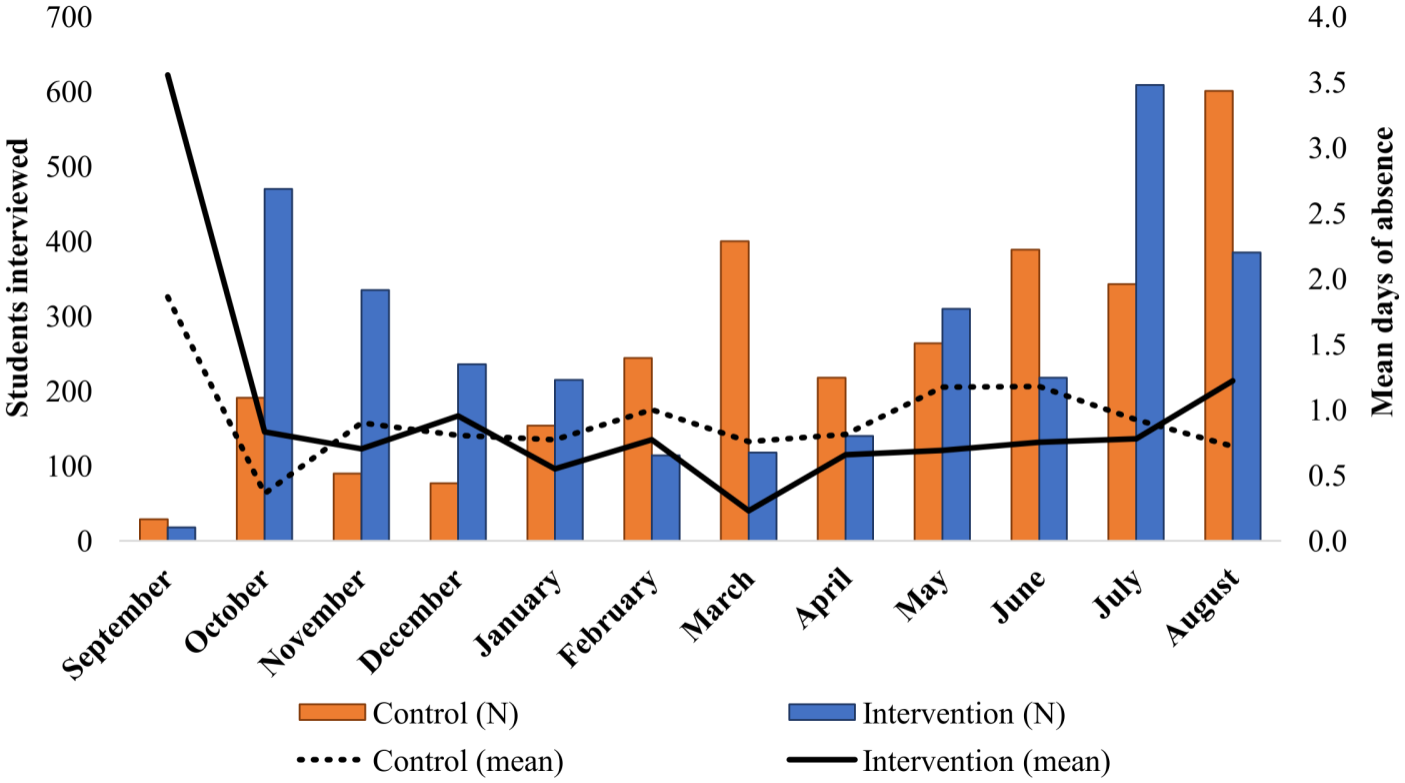
Number of students interviewed and mean days of absence, by month and trial group.

Fig 3 shows the distribution of absence days in the past four weeks among students in the ITT sample, measured in the first HDSS survey after CAPS enrolment, by cookstove status. Overall, students in the intervention group missed an average of 0.81 days in the past four weeks, relative to 0.88 days in the control group. Similar proportions of students in each arm—26.9% intervention, 27.8% control—missed one or more days of school, and among those who were absent, the mean number of days missed was also very similar across groups: 3.0 among intervention students, 3.1 among controls. Fig 4 charts the mean days of absence by month of interview among intervention and control students, as well as the total number of students interviewed in each month. With the exception of a sharp peak in September when very few students were interviewed, rates of absenteeism were fairly flat across the school year, and consistently higher in the control group over the period January-June. As a result of the clustered nature of data collection, the distribution of interviews across the school year varied across trial groups; as such, multivariable regression models adjust both for a student’s month of interview, as well as whether he/she was interviewed outside of term time.

Results from negative binomial regression models suggest that, although the rate of absenteeism was slightly lower among cookstove recipients, there was no evidence that the CAPS trial yielded measurable improvements in school attendance in either crude or adjusted analyses (adjusted incidence rate ratio (IRR) 0.92 [95% confidence interval (CI) 0.71–1.18]; Table 2). The per-protocol analysis gave similar results (adjusted IRR 0.93 [0.71–1.23]). Alternative specifications of the per-protocol sample—such as excluding only households that reported not using the cookstove at all, or only those that reported continuing to use open fire cooking methods for every, rather than any, household meal—did not change the nature of these findings.

**Table 2:** Unadjusted and adjusted incidence rate ratios of absenteeism in the past four weeks, comparing intervention to control group.

	Unadjusted IRR	95% CI	p-value	Adjusted IRR	95% CI	p-value
ITT sample (N = 6168)	0.92	0.72–1.19	0.53	0.92	0.71–1.18	0.51
Per-protocol sample (N = 4784)	0.93	0.71–1.22	0.60	0.93	0.71–1.23	0.61

Notes: Results from negative binomial regression models with cluster robust standard errors. IRR = incidence rate ratio; CI = confidence interval. Adjusted model also includes: age, sex, current grade attended, repetition of current grade, maternal death, paternal death, maternal education, paternal education, number of total household members, number of younger household members, relationship to household head, sex of household head (taken from HDSS schooling survey), household socioeconomic status, coresidence with a regular smoker, exposure to sources of household smoke other than cooking (taken from CAPS baseline survey), the month of HDSS interview, HDSS survey round, interview timing relative to school holidays, and months between CAPS enrolment and HDSS survey. The intra-class correlation was estimated at 0.04.

The stratified analyses presented in Table 3 investigate the relationship between cookstove status and absenteeism by sex, age, and season. Although the adjusted IRRs showed absenteeism reductions in the hypothesised groups—namely, female cookstove recipients, students in the older age categories, and students interviewed during the rainy season—no conclusive evidence for effect modification was found. To investigate whether combining these subgroups yielded cookstove-related attendance benefits, separate models were run for girls and boys among students interviewed during the rainy season. These did provide some evidence that the cookstoves were associated with reduced absenteeism among girls aged 15 or older (Table 4), but this category comprised relatively few students.

**Table 3:** Unadjusted and adjusted stratified analysis of the relationship between cookstove exposure and days of absence in the past four weeks, by sex, age and season of interview.

	Unadj. IRR	95% CI	p-value	p-value interaction	Adj. IRR	95% CI	p-value	p-value interaction
**ITT sample (N = 6168)**								
**1) By sex**				0.52				0.40
Male (N = 3157)	0.96	0.73–1.27	0.77		0.97	0.74–1.27	0.82	
Female (N = 3011)	0.88	0.67–1.17	0.39		0.87	0.64–1.17	0.35	
**2) By age (years)**				0.73				0.89
<12 (N = 4155)	0.92	0.72–1.19	0.54		0.94	0.72–1.21	0.62	
12–14 (N = 1310)	0.87	0.61–1.22	0.41		0.88	0.65–1.20	0.43	
≥15 (N = 703)	1.03	0.63–1.66	0.91		0.88	0.55–1.40	0.60	
**3) By season**				0.58				0.55
Dry (N = 4252)	0.95	0.73–1.24	0.71		0.97	0.75–1.25	0.80	
Rainy (N = 1916)	0.81	0.48–1.35	0.41		0.81	0.48–1.37	0.43	
**Per-protocol sample (N = 4784)**								
**1) By sex**				0.40				0.23
Male (N = 2456)	0.98	0.73–1.32	0.92		1.01	0.76–1.37	0.90	
Female (N = 2328)	0.86	0.62–1.20	0.38		0.84	0.60–1.18	0.32	
**2) By age (years)**				0.43				0.71
<12 (N = 3220)	0.93	0.71–1.22	0.61		0.95	0.71–1.27	0.74	
12–14 (N = 1015)	0.81	0.52–1.25	0.34		0.84	0.57–1.23	0.36	
≥15 (N = 549)	1.18	0.68–2.03	0.55		1.00	0.59–1.68	0.99	
**3) By season**				0.38				0.35
Dry (N = 3179)	0.99	0.75–1.32	0.96		0.99	0.74–1.33	0.96	
Rainy (N = 1605)	0.75	0.43–1.31	0.31		0.73	0.41–1.30	0.29	

Notes: Stratum-specific IRRs from three negative bionomial regression models with cluster robust standard errors and interactions between cookstove status and 1) sex, 2) age group, and 3) season. IRR = incidence rate ratio; CI = confidence interval. All adjusted models include the covariates listed in Table 2, with the exception of Model 3, which excludes month of interview due to collinearity with interview season. Wald tests were used to assess evidence for interaction.

**Table 4:** Unadjusted and adjusted stratified analysis of the relationship between cookstove exposure and days of absence in the past four weeks among boys and girls interviewed during the rainy season, by age.

	Unadj. IRR	95% CI	p-value	p-value interaction	Adj. IRR	95% CI	p-value	p-value interaction
**Boys: ITT sample (N = 996)**								
**Overall**	0.75	0.46–1.24	0.27	--	0.62	0.39–0.99	0.045	--
**By age**				0.88				0.44
<12 (N = 669)	0.78	0.47–1.29	0.33		0.63	0.40–0.99	0.05	
12–14 (N = 200)	0.64	0.27–1.48	0.29		0.47	0.23–0.94	0.03	
≥15 (N = 127)	0.74	0.25–2.22	0.60		0.89	0.29–2.75	0.84	
**Boys: Per-protocol sample (N = 834)**								
**Overall**	0.72	0.40–1.29	0.27	--	0.59	0.35–0.98	0.04	--
**By age**				0.67				0.27
<12 (N = 557)	0.74	0.41–1.36	0.34		0.59	0.35–0.99	0.046	
12–14 (N = 168)	0.48	0.17–1.41	0.18		0.35	0.13–0.92	0.03	
≥15 (N = 109)	0.83	0.25–2.70	0.76		1.04	0.33–3.35	0.94	
**Girls: ITT sample (N = 920)**								
**Overall**	0.87	0.46–1.65	0.67	--	0.68	0.38–1.24	0.21	--
**By age**				0.04				0.12
<12 (N = 664)	1.11	0.57–2.16	0.77		0.79	0.43–1.46	0.45	
12–14 (N = 182)	0.52	0.24–1.14	0.10		0.58	0.26–1.33	0.20	
≥15 (N = 74)	0.46	0.17–1.21	0.11		0.32	0.14–0.75	0.009	
**Girls: Per-protocol sample (N = 771)**								
**Overall**	0.79	0.41–1.52	0.48	--	0.71	0.39–1.28	0.26	--
**By age**				<0.001				0.14
<12 (N = 565)	1.05	0.54–2.03	0.89		0.88	0.47–1.64	0.69	
12–14 (N = 149)	0.41	0.17–1.00	0.050		0.51	0.21–1.27	0.15	
≥15 (N = 57)	0.31	0.06–1.62	0.16		0.20	0.04–1.02	0.053	

Notes: Pooled and stratum-specific IRRs from negative bionomial regression models with cluster robust standard errors, among girls and boys interviewed during the rainy season. Stratum-specific IRRs generated by interacting cookstatus and age group. IRR = incidence rate ratio; CI = confidence interval. All adjusted models include the covariates listed in Table 2. Wald tests were used to assess evidence for interaction

### Reasons for absenteeism

**Fig 5:**
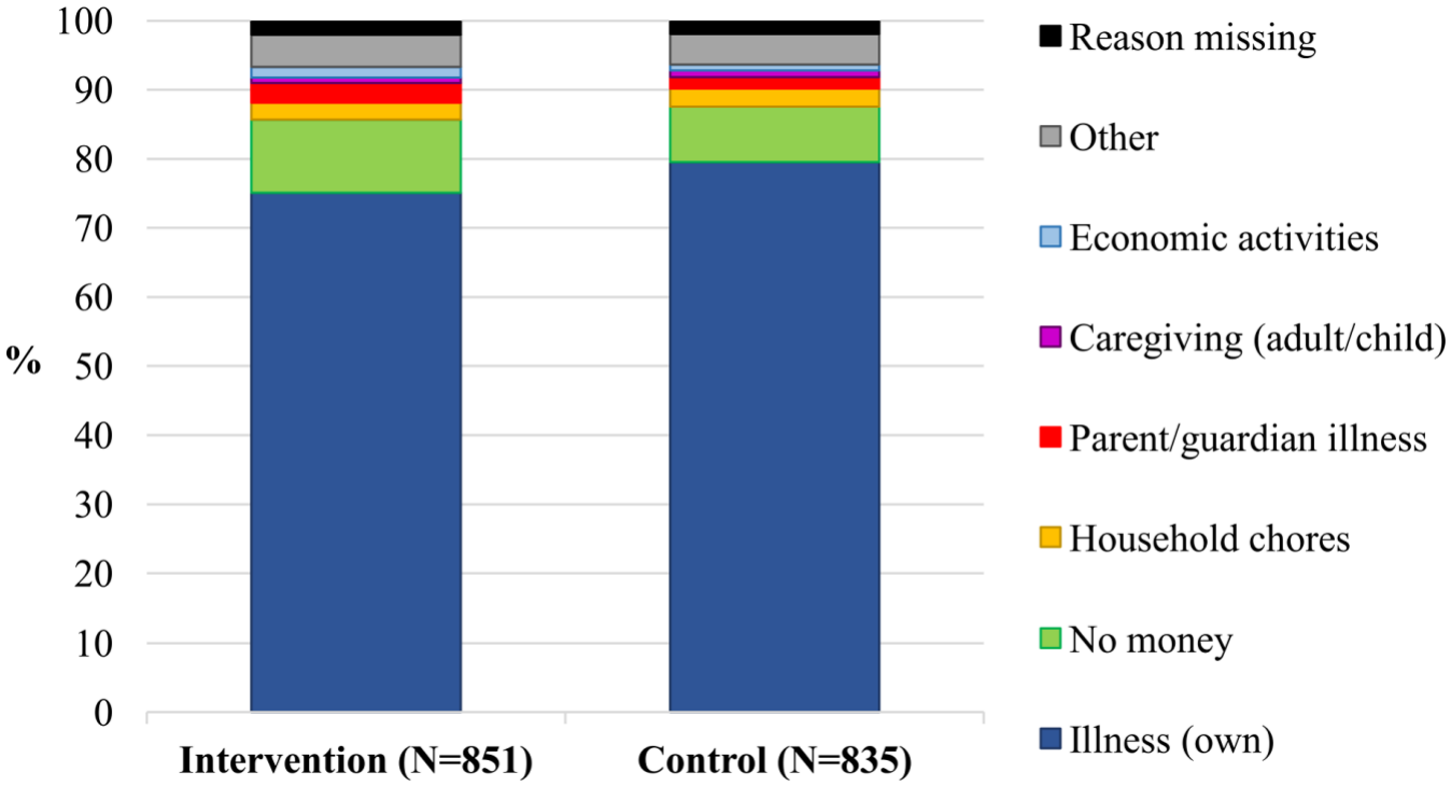
Primary reason reported for missed school in the past four weeks, by trial group.

Fig 5 shows that, among members of the ITT sample who were absent in the past four weeks, no difference in the distribution of reasons reported for missing school was found between the intervention and control groups. In both cases, the vast majority of absences (75.1% in the intervention group, 79.5% in the control group) were attributed to illness, while household chores, economic activities and caregiving were reported to make consistently negligible contributions to absenteeism. Although no school fees are charged for primary education in Malawi, approximately 10% of absences (10.6% intervention, 8.0% control) were attributed to lack of money for school supplies, transport or meals.

### Qualitative analysis

Data from IDIs and FGDs confirmed that ill health represented an important cause of school absence. However, many of the sicknesses described would not be expected to derive from HAP exposure, such as malaria and stomach ache. Indeed, most participants did not perceive any change in household health status resulting from the cookstoves: “Sickness is sickness, it just comes” (Male, intervention group (IG)). One IDI participant did observe, however, that her sister suffered fewer asthma attacks since receiving the cookstoves—“[She] used to be sick but now has changed…Doesn’t get sick often nowadays” (Female, IG)—which may have had positive implications for school attendance.

Although the perceived health benefits of the cookstoves were less pronounced than anticipated, there was nearly universal agreement among students from intervention households that the cookstoves produced time and resource savings. Many participants observed that the cookstoves used considerably less fuel than traditional open fire cooking, which reduced fuel collection burdens:

On three stones [open fire] methods we used more firewood but now we use little firewood. (Female, IG)

When using little firewood, it doesn’t take time to go and fetch for the firewood. (Female, IG)

Because the cookstoves needed only small pieces of wood and biomass, which could be readily found, another participant noticed time savings from not needing to chop firewood:

For three stone method, it requires you to first put the stones but when using a cookstove you just insert the small pieces of firewood and set fire. For three stone method, you need also to take an axe and cut firewood which is a waste of time. (Male, IG)

Participants also agreed that cooking meals was noticeably quicker when using the cookstoves, partly because households were given two units and cooking pots and so could prepare multiple dishes at once, but also because the cookstove fire burned much more efficiently: “When cooking using the cookstove, the food cooks fast… Because when using the cookstove, the fire goes direct to the pot unlike when using three stones the fire gets wasted” (Female, IG).

While these insights provide support for the hypothesis that the cookstoves yielded important time and resource savings, it is less clear that these savings translated into reduced school absenteeism. In particular, evidence that cooking or resource collection interfered with daily school attendance was limited, even in the control group. Among students, primarily girls, who reported collecting fuelwood, all described doing so on weekends or holidays, or during free time after school, such that it was compatible with school attendance:

**Interviewer (I)**: … **[D]o you ever fetch firewood?**

Participant (P): Yes but I usually do this on Saturdays.

**I**: … **Have you ever missed school because of fetching firewood?**

P: I have never since we fetch firewood on Saturdays when we don’t go to school. (Female, control group (CG))

**I**: **So when maybe you are fetching maize cobs, how long do you take?**

P: I don’t take time, maybe only one hour. (Female, CG)

A minority of participants indicated that their households purchased wood or charcoal for cooking, but did not comment on any changes in expenditure as a result of the cookstoves.

As anticipated, responsibility for cooking itself was borne predominantly by female household members, and often discussed in gendered terms. One male focus group participant observed, for example, that: “Girls can sometimes be told to miss school so that they should just cook food when we [boys] are at the farm and when we come back from the farm, we should find that the food is already cooked” (Male, CG). Among female participants, however, cooking was portrayed as largely compatible with school attendance, with students taking responsibility for the afternoon or evening meals upon return from school. As such, cooking-related absences were rarely reported, although one FGD participant described missing school to help with other aspects of food preparation: “If maybe at home they are expecting visitors, they say don’t go to school, you have to chase that chicken for visitors who are coming” (Female, IG). None of the cookstove recipients linked the cookstove to any changes in school attendance.

Instead, students reported a number of barriers to school attendance that were unrelated, either directly or indirectly, to cooking activities. In addition to ill health, these included engaging in household agricultural work or informal paid labour to help raise funds for schooling expenses, or school-related issues such as lack of uniform or supplies. There was some suggestion, however, that the cookstoves improved other educational outcomes not captured by our measure of school attendance. For instance, two participants observed that reduced time to cook breakfast in the morning resulted in fewer late arrivals to school:

I go early to school when I cook on new cookstoves rather than on three stone cookstoves which requires more time to prepare fire. (Male, IG)

**I**: **What is the change that you have noticed about time you go to school?**

P: We cook food very fast and eat earlier and then we rush to school.

**I**: **So you mean you go to school early?**

P: Yes. (Female, IG)

Even when cooking or fuelwood collection does not directly compete with schooling, reduced time burdens associated with these activities may allow students to spend more time studying or resting, with positive implications for school performance. Although most IDI participants reported having adequate time to combine household work and self-study, when asked a hypothetical question about how they would use any time savings from reduced domestic burdens, 11 of 16 indicated that they would spend the extra time on reading, writing or homework, including the following students:

**I**: **Do you have enough time in your day to spend on school and homework here at home?**

P: Yes.

**I**: **If you could spend less time on doing household chores, how would you use the extra time in your day?**

P: I can use it to read my school notes and also revising what I got wrong at school.

**I**: **Is there any other thing you can also do?**

P: Apart from that, I can also be doing my homework…

**I**: **Suppose you have done your homework but still more you have extra time, how would you use it?**

P: Then I can be drawing water in preparation for tomorrow. (Female, IG)

**I**: **Do you have enough time in your day to spend on school and homework?**

P: Yes.

**I**: **If you could spend less time on doing household chores, how would you use the extra time in your day?**

P: I can use it for reading.

**I**: **Why reading?**

P: *(Silence)*

**I**: **Is there anything else you could do apart from reading?**

P: After reading if I still have time then I can use it to cook.

**I: Suppose you have finished cooking**.

P: Then to play with my friends. (Female, CG)

## Discussion

This study combined quantitative and qualitative data to assess the impact of cleaner burning biomass-fuelled cookstoves on primary school attendance in northern Malawi and found that the CAPS intervention had no measurable impact on primary school attendance overall. These findings echo a separate evaluation of the CAPS trial’s primary outcome, which found no evidence that the cookstoves reduced the incidence of pneumonia in children under five years old [20]. The authors of the latter study speculated that the cookstoves did not sufficiently reduce exposure to air pollution in a context where other forms of smoke exposure including burning rubbish, brick burning and tobacco smoking were prevalent [20].

The target population of the CAPS trial consisted of children under five years old, so data were not collected from school-age household members with which to assess the direct impact of the cookstoves on adolescent health. Most participants in the qualitative study did not perceive a change in household health after receiving the cookstoves, although there was some suggestion that the cookstoves reduced asthma exacerbations. Strong links have been drawn between open fire cooking and asthma prevalence in both younger (age 6–7) and older (age 13–14) school-age children in global studies [7], but asthma is reported to be uncommon among children in Malawi, comprising just 0.6% of cases admitted to hospital [28]. Although the CAPS evaluation found that the cookstoves significantly reduced burns among children under five [20], it is likely that students’ caregiving responsibilities did not diminish to a sufficient degree to observe a population-level impact on school attendance.

Qualitative data confirmed that students perceived time and resource savings associated with the cookstoves, consistent with findings from a CAPS socioeconomic study carried out among primary cooks [29]. Evidence that these savings translated into improvements in school attendance, however, was minimal. In particular, participants from both trial groups indicated that cooking-related responsibilities were compatible with school attendance. Wodon and Beegle [30], using data from the 2004 Malawi Second Integrated Household Survey, examined the contribution of various activities to household labour and found that the time associated with fuelwood collection—between 0.1 and 0.5 hours per week for rural boys aged 5–14 and 0.4–1.2 hours for rural girls, depending on the month—was relatively limited, suggesting that it could be successfully combined with schooling. As reflected in our qualitative findings, larger time burdens were associated with agricultural labour and, for girls, also with other household chores including cooking, laundry, cleaning, and water collection [30].

While the primary analysis followed ITT principles, the per-protocol analysis excluded students from households that did not report using the cookstove exclusively during the follow-up period corresponding to the schooling survey. Three percent of students lived in households in which the cookstove was not used at all, while a further 33% came from households that continued using open fire cooking methods alongside the cookstove. Non- and concurrent use of cookstoves has been noted in cookstove trials in a variety of settings [31–34] and highlights the difficulty of implementing interventions involving behaviour change. Even employing a strict per protocol definition there was no evidence of benefit of the cookstoves on absenteeism, with the possible exception of older girls interviewed during the rainy season. The latter finding merits further research.

### Limitations

Several limitations to the present study should be noted. Firstly, because CAPS cookstoves were offered only to households with children under five, findings are representative only of households in the catchment area with both young and school-age children. Secondly, the sample size for some subgroup analyses was limited, particularly those presented in Table 4, so these results should be interpreted with caution.

The study was additionally limited by the range of data available. In particular, it lacked detailed time use information from the larger survey sample with which to quantify the time burdens attached to fuel collection and cooking among household members in control and intervention clusters, to identify other activities that inhibit school attendance, and to establish the extent to which students complete homework or engage in non-school-related educational activities such as reading or listening to the radio. Hourly, rather than daily, school attendance data would also have enabled an examination of the cookstoves’ impact on a more nuanced set of school attendance outcomes, including timely arrival at school, which the qualitative data suggest may have improved in intervention households.

Finally, although insights from IDIs and FGDs shed valuable light on the proposed mechanisms (or lack thereof) linking cleaner burning cookstoves and school absenteeism, the scope of the qualitative study was limited by the time and resources available. A pre-/post-intervention interview design, as well as a larger sample size and a more explicit focus on seasonality, would have provided greater depth, particularly with respect to changing perceptions surrounding health, time use, and schooling.

## Conclusion

This mixed methods study combined quantitative and qualitative data to assess the impact of cleaner burning cookstoves on primary school absenteeism in Karonga district, northern Malawi. Taken together, the findings indicate that the cookstoves did not yield measurable reductions in primary school absenteeism, but suggest that they might confer other school-related benefits not captured by the outcome measure available. On this evidence, interventions that aim to increase school participation should more directly target the barriers to school attendance that are salient in this population, including cost constraints and non-HAP-related illness. An appreciation of context is important, however: Malawi’s northern region, where the study was located, is characterised both by comparatively favourable educational outcomes [35, 36], as well as higher forest cover than the two southerly regions [37]. Interventions from settings where rates of absenteeism are higher and fuelwood more scarce may yield different results.
